# Supplementary material for: Charged Amino Acids in the Transmembrane Helix Strongly Affect the Enzyme Activity of Aromatase
Source: Int J Mol Sci. 2024 Jan 24;25(3):1440. doi: 10.3390/ijms25031440 (PMC10855386; doi:10.3390/ijms25031440)
Supplement: Supplementary file 1 [file ijms-25-01440-s001.zip › Figure S1.pdf]

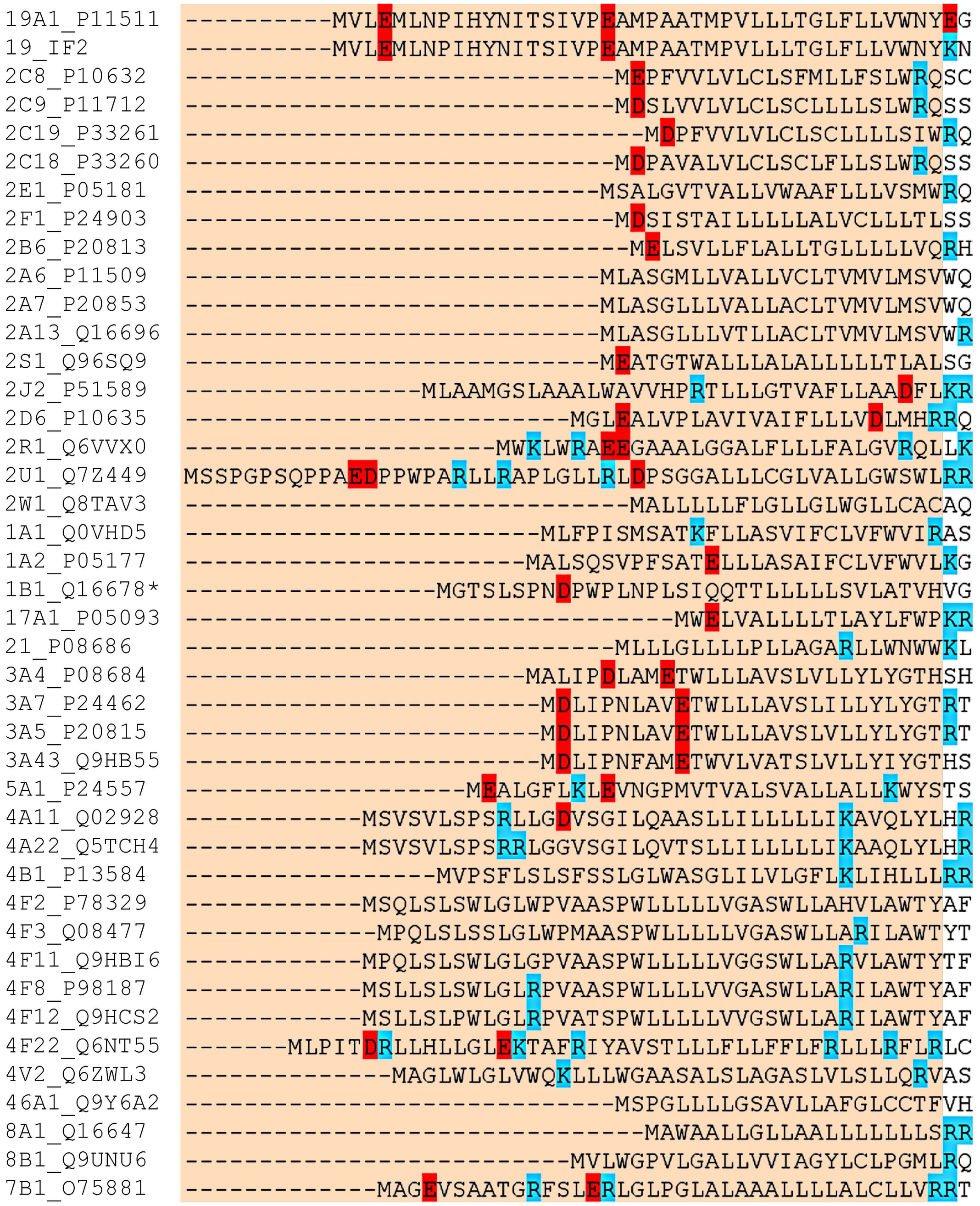

Figure S1. Membrane-embedded residues of the N-termini of 41 human microsomal CYP450 proteins and the CYP19 KN-TY mutant (19_IF2). Calculations were performed with the web tool PPM 3.0 [1] using non-curved mammalian ER membranes and their physicochemical and mechanical properties. The analyses were based on the respective AlphaFold structures of the CYP450 proteins. The non-cytoplasmically exposed amino acids of the N-terminus are marked by a beige box. Additionally, the first two amino acids located in the cytoplasm are indicated. Acidic amino acids E and D are highlighted in red, basic amino acids K and R are highlighted in blue. The names of the corresponding CYP450 proteins and the UniProt ID of the AlphaFold pdb structures used are shown. Jalview 2.11.2.7 [2] was used to visualize the amino acid sequences. *CYP1B1; no transmembrane secondary structure segment could be calculated by the program, but the amino acids marked in beige could be calculated as embedded in the membrane.

1. Lomize, A.L.; Todd, S.C.; Pogozheva, I.D. Spatial arrangement of proteins in planar and curved membranes by PPM 3.0. *Protein Science* **2022**, *31*, 209-220, doi:https://doi.org/10.1002/pro.421
2. Waterhouse, A.M.; Procter, J.B.; Martin, D.M.A.; Clamp, M.; Barton, G.J. Jalview Version 2—a multiple sequence alignment editor and analysis workbench. *Bioinformatics* **2009**, *25*, 1189-1191, doi:10.1093/bioinformatics/btp033.
