# Supplementary material for: Charged Amino Acids in the Transmembrane Helix Strongly Affect the Enzyme Activity of Aromatase
Source: Int J Mol Sci. 2024 Jan 24;25(3):1440. doi: 10.3390/ijms25031440 (PMC10855386; doi:10.3390/ijms25031440)
Supplement: Supplementary file 1 [file ijms-25-01440-s001.zip › Figure S2.pdf]

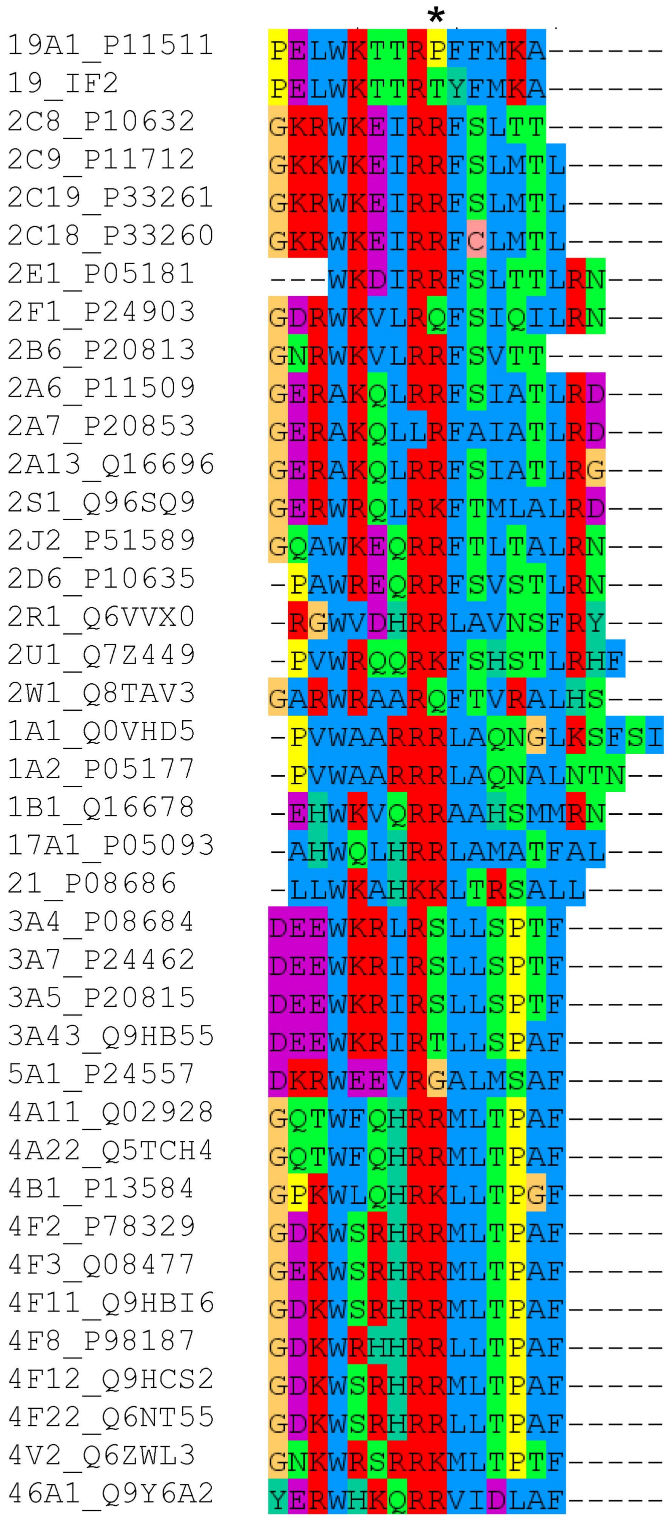

Figure S2. Amino acid sequences of helix C of 38 human CYP450 proteins and isoform 2 of aromatase (19_IF2). The amino acids are colored according to the Clustral X code. The names and UniProt IDs of the CYP450 proteins are given. CYP7 and CYP8 proteins form a separate group of atypical CYP450 enzymes. Their protein structure lacks the typical helix C. Jalview 2.11.2.7 [1] was used to visualize the amino acid sequences. *Proline in the middle of helix C of aromatase isoform 1.

1. Waterhouse, A.M.; Procter, J.B.; Martin, D.M.A.; Clamp, M.; Barton, G.J. Jalview Version 2—a multiple sequence alignment editor and analysis workbench. *Bioinformatics* **2009**, *25*, 1189-1191, doi:10.1093/bioinformatics/btp033.
