# Supplementary material for: Charged Amino Acids in the Transmembrane Helix Strongly Affect the Enzyme Activity of Aromatase
Source: Int J Mol Sci. 2024 Jan 24;25(3):1440. doi: 10.3390/ijms25031440 (PMC10855386; doi:10.3390/ijms25031440)
Supplement: Supplementary file 1 [file ijms-25-01440-s001.zip › Supplementary File S1.pdf]

Supplementary File S1

Charged amino acids in the transmembrane helix strongly affect the enzyme activity of aromatase

Juliane Günther, Gerhard Schuler, Elin Teppa, Rainer Fürbass

Supplementary Data. Protein sequences of Artiodactyla species used for the identification of the two aromatase isoforms. Annotated proteins are listed as sequences, including the NCBI protein accession numbers. Protein sequences identified in this study using the Blastn search in RefSeq genome databases or whole-genome shotgun contigs are indicated with both the sequence and position of the corresponding coding exons.

Catagonus wagneri

>CYP19A1 (incomplete)
MVTVCADSITRHLDKLEEVRNDLGYVDVLTLMRRIMLDASNNLFLGIPLESSSTFHIMKHSHYTSRFGSKLGLECIGMHEKGIIFNNNP
ALWKAVRPFFIKGPGYFLGIGPLISHFRFLWMGIGSACNYYNKMYGEFTRVWIGGEETLIISRLNIAFPEIKQYKMVLEMLNPMHYNII
TNMVSEAVPLASIAILLLTGFLLWVWNYEDTSSIP…exon5,6,7_missing…ERDIRNDDMHKLKVVENVIYESMRYQPVVDLVMRK
ALEDDVIDGYPVKKGTNIILNIGRMHRLEFFPKPNEFTLENFAKNVPHRYFQPFGFGPRACAGKFIAMVMMKVTVVTLLRRFQVQTQKD
WCIEKMKKKNDLSSHPDETRGLLEMIFIPRN_DKCLEH
Catagonus wagneri isolate BS18 Sc2sB16_1707;HRSCAF=42189_2, whole genome shotgun sequence
Exon 1 >PVHT020001751.1:14637099-14637284
Exon 2 >PVHT020001751.1:14631526-14631675
Exon 3 >PVHT020001751.1:14617898-14618052
Exon 4 >PVHT020001751.1:14615229-14615404
Exon 5, 6, 7 missing
Exon 8 >PVHT020001751.1:14577507-14577748
Exon 9 >PVHT020001751.1:14578930-14579962
>CYP19A2 (incomplete)
MVSEMLNPVHYKITSMVSEVVPFASTAVVILTGFLLLVWNYGNISSIPGPGYFLGIGPLISYFRFLWMGIGSACNYYNKMYGEFMRVWI
GGEETLIISNSHKISSNNFPEDWIVVKYDAFLMHSNELQPRFAAESGCVVTVLHYMEHT_GPSNGIPRKRLLEASSMMRRMSVNTST_P
KSLRNSSNLSRCLVVESAQTVTMRARPGPDREKAIVCKIQGYFDAWQALLLKPDIFFKIPWLYRKYEKSVKELKEDVEILTEKKRRRVF
IAEKLESCMDFATELILAEKRGELTRENVNQCVLEMLIAAPDTMSVSVFFMLFLIAKHPQVEEELMKEIQTVV…exon8_missing…V
PYRYFQPFGFGPRACAGKYIAMVMMKVILVTLLRRFQVQTLQDRCVEKMQKKNDLSLHPDETRGLLEMIFIPRNSDKCFTK
Catagonus wagneri isolate BS18 Sc2sB16_1707;HRSCAF=42189_2, whole genome shotgun sequence
Exon 1 >PVHT020001751.1:14568370-14568551
Exon 2 >PVHT020001751.1:14571424-14571574
Exon 3 >PVHT020001751.1:14551785-14551939
Exon 4 >PVHT020001751.1:14546785-14546961
Exon 5 >PVHT020001751.1:14539058-14539172
Exon 6 >PVHT020001751.1:14541730-14541844
Exon 7 >PVHT020001751.1:14542632-14542794
Exon 8 missing
Exon 9 >PVHT020001751.1:14536004-14536929
>CYP19A3
MVSEMLNPVHYKITSMVSEVVPFASTAVVILTGFLLLVWNYGNISSIPGPGYFLGIGPLISYFRFLWMGIGSACNYYNKMYGEFMRVWI
GGEETLIISKSSSVFHVMKHSHYTSRFGSKPGLQFIGMHEKGIIFNNNPVLWKVVRTYFMRALSGPGLARMVTVCADSTTRHLDKLEEF
RNDLGYVDVLTLMRRIMLDASNNLFLGIPLDEKAIVCKIQGYFDAWQALLLKPDIFFKIPWLYRKYEKSVKELKEDVEILTEKKRRRVF
IAEKLESCMDFATELILAEKRGELTRENVNQCVLEMLIAAPDTMSVSVFFMLFLIAKHPQVEEELMKEIQTVVGERDIRNDDMHKLKVV
ENVIYESMRYQPVVDLVMRKALEDDVIDGYPVKKGTNIILNIGRMHRLEFFPKPNEFTLENFAKNVPYRYFQPFGFGPRACAGKYIAMV
MMKVILVTLLRRFQVQTLQDRCVEKMQKKNDLSLHPDETRGLLEMIFIPRNSDKCFTK

Catagonus wagneri isolate BS18 Sc2sB16_1707;HRSCAF=42189_2, whole genome shotgun sequence
Exon 1 >PVHT020001751.1:14568370-14568551
Exon 2 >PVHT020001751.1:14571424-14571574
Exon 3 >PVHT020001751.1:14551785-14551939
Exon 4 >PVHT020001751.1:14546785-14546961
Exon 5 >PVHT020001751.1:14539058-14539172
Exon 6 >PVHT020001751.1:14541730-14541844
Exon 7 >PVHT020001751.1:14542632-14542794
Exon 8 >PVHT020001751.1:14577507-14577748
Exon 9 >PVHT020001751.1:14536004-14536929

Phacochoerus africanus

>XP_047622306.1 aromatase 3 [Phacochoerus africanus]
MVLEMLNPMNISSMVSEAVLFGSIAILLLIGLLLWVWNYEGTSSIPGPGYFLGIGPLISHFRFLWMGIGSACNYYNKMYGEFMRVWIGG
EETLIISKSSGMFHVMKHSHYTCRFGSKLGLECIGMHEKGIMFNNNPALWKAVRPFFTKALSGPGLVRMVTVCADSITKHLDKLEEVRN
DLGYVDVLTLMRRIMLDTANNLFLGIPLDESALVDKVQGYFDAWQALLLKPDIFFKISWLYRKYEKSVKDLKDAMDILIEEKRHRISTA
EKLEDSMDFTTQLIFAEKRGELTKENVNQCVLEMLIAAPDTMSITVFFMLFLIANHPKVEEELMKEIYTVVGERDIRNDDMQKLKVVEN
FIYESMRYQPVVDLVMRKALEDDVIDGYPVKKGTNIILNIGRMHRLEFFPKPNEFTLENFAKNVPYRYFQPFGFGPRACAGKYIAMVMM
KVILVTLLRRFQVQTQQGQCVENLEKKNDLSLHPDETSGLLEMIFIPRNSDKCLEH
>XP_047622307.1 aromatase 1 [Phacochoerus africanus]
MVLEMLNPMHYKVTSMVSEVVPFASIAVLLLTGFLLLVWNYKNTSSIPGPGYFLGIGPLISYLRFLWMGIGSACNYYNKTYGEFIRVWI
GGEETLIISKSSSVFHVMKHSHYTSRFGSKPGLQFIGMHEKGIIFNNNPVLWKAVRTYFMKALSGPGLVRMVTVCADSITKHLDKLEEV
RNDLGYVDVLTLMRRIMLDTANNLFLGIPLDEKAIVCKIQGYFDAWQALLLKPDIFFKIPWLYRKYEKSVRYLKEDMEILIEKKRRRIF
TAEKLEDCMDFATELILAEKRGELTKENVNQCILEMLIAAPDTMSVTVFFMLFLIAKHPQVEEELMKEIQTVVGERDIRNDDMQKLEVV
ENFIYESMRYQPVVDLVMRKALEDDVIDGYPVKKGTNIILNIGRMHRLEFFPKPNEFTLENFAKNVPYRYFQPFGFGPRACAGKYIAMV
MMKVTLVILLRRFQVQTPQDRCVEKMQKKNDLSLHPDETNGLLEMIFIPRNSDKCFTK
>XP_047622308.1 aromatase 2 [Phacochoerus
africanus]MVLEMLNPMYYKITSMVSEVVPFASIAVLLLTGFLLLLWNYENTSSIPGPGYFLGIGPLISHFRFLWMGIGSACNYYNK
MYGEFMRVWIGGEETLIISKSSSVFHVMKHSHYTSRFGSKPGLECIGMYEKGIIFNNDPALWKAVRTYFMKALSGPGLVRMVTVCADSI
TKHLDKLEEVRNDLGYVDVLTLMRRIMLDTSNNLFLGIPLDEKTIVCKIKSYFDAWQALLLKPEFFFKFSWLYKKHKESVKDLKESMEI
LIEKKRCSIITAEKLEDCMDFATELILAEKRGELTKENVNQCILEMLIAAPDTLSVTVFFMLFLIAKHPQVEEAIVKEIQTVIGERDIR
NDDMQKLKVVENFIYESMRYQPVVDLVMRKALEDDVIDGYPVKKGTNIILNIGRMHRLEFFPKPNEFTLENFAKNVPYRYFQPFGFGPR
ACAGKYIAMVMMKVTLVILLRRFQVQTPQDRCVEKMQKKNDLSLHPDETSGLLEMIFIPRNSDKCFTK

Sus scrofa

>NP_999596.1 aromatase 3 [Sus scrofa]
MVLEMLNPMNISSMVSEAVLFGSIAILLLIGLLLWVWNYEDTSSIPGPGYFLGIGPLISHFRFLWMGIGSACNYYNKMYGEFMRVWIGG
EETLIISKSSSIFHIMKHNHYTCRFGSKLGLECIGMHEKGIMFNNNPALWKAVRPFFTKALSGPGLVRMVTVCADSITKHLDKLEEVRN
DLGYVDVLTLMRRIMLDTSNNLFLGIPLDESALVHKVQGYFDAWQALLLKPDIFFKISWLYRKYEKSVKDLKDAMEILIEEKRHRISTA
EKLEDSMDFTTQLIFAEKRGELTKENVNQCVLEMMIAAPDTMSITVFFMLFLIANHPQVEEELMKEIYTVVGERDIRNDDMQKLKVVEN
FIYESMRYQPVVDFVMRKALEDDVIDGYPVKKGTNIILNIGRMHRLEFFPKPNEFTLENFAKNVPYRYFQPFGFGPRACAGKYIAMVMM
KVILVTLLRRFQVQTQQGQCVEKMQKKNDLSLHPHETSGLLEMIFIPRNSDKCLEH
>NP_999594.1 aromatase 1 [Sus scrofa]
MVLEMLNPMHYKVTSMVSEVVPFASIAVLLLTGFLLLVWNYKNTSSIPGPGYFLGIGPLISYLRFLWMGIGSACNYYNKTYGEFIRVWI
GGEETLIISKSSSVFHVMKHSHYTSRFGSKPGLQFIGMHEKGIIFNNNPVLWKAVRTYFMKALSGPGLVRMVTVCADSITKHLDKLEEV
RNDLGYVDVLTLMRRIMLDTSNNLFLGIPLDEKAIVCKIQGYFDAWQALLLKPDIFFKIPWLYRKYEKSVKDLKEDMEILIEKKRRRIF
TAEKLEDCMDFATELILAEKRGELTKENVNQCILEMLIAAPDTMSVTVFFMLFLIAKHPQVEEELMKEIQTVVGERDIRNDDMQKLEVV
ENFIYESMRYQPVVDLVMRKALEDDVIDGYPVKKGTNIILNIGRMHRLEFFPKPNEFTLENFAKNVPYRYFQPFGFGPRACAGKYIAMV
MMKVTLVILLRRFQVQTPQDRCVEKMQKKNDLSLHPDETSGLLEMIFIPRNSDKCFTK
>NP_999595.1 aromatase 2 [Sus scrofa]
MVLEMLNPMYYKITSMVSEVVPFASIAVLLLTGFLLLLWNYENTSSIPSPGYFLGIGPLISHFRFLWMGIGSACNYYNEMYGEFMRVWI
GGEETLIISKSSSVFHVMKHSHYTSRFGSKPGLECIGMYEKGIIFNNDPALWKAVRTYFMKALSGPGLVRMVTVCADSITKHLDKLEEV
RNDLGYVDVLTLMRRIMLDTSNNLFLGIPLDEKAIVCKIQGYFDAWQALLLKPEFFFKFSWLYKKHKESVKDLKENMEILIEKKRCSII
TAEKLEDCMDFATELILAEKRGELTKENVNQCILEMLIAAPDTLSVTVFFMLFLIAKHPQVEEAIVKEIQTVIGERDIRNDDMQKLKVV
ENFIYESMRYQPVVDLVMRKALEDDVIDGYPVKKGTNIILNIGRMHRLEFFPKPNEFTLENFAKNVPYRYFQPFGFGPRACAGKYIAMV
MMKVTLVILLRRFQVQTPQDRCVEKMQKKNDLSLHPDETSGLLEMIFIPRNSDKSLDH

Camelus dromedarius

>XP_031308923.1|:1-507 LOW QUALITY PROTEIN: aromatase [Camelus dromedarius]
MVLEMLNPIHYYNITSMVSGGVPVAAMAILLLTGFLLLVWNYEDTSSIPGPGYCLGIGPLISHCRFLWMGIGSASNYYNKMYGEFMRVW
ICGEETLIISKSSSMFHVMKHSHYISRFGSKLGLQFIGMHEKGIIFNNNPALWKAVRPFFTKALSGPGLVRMVTVCADSITKHLDRLEE
VRDEMGYVDVLTLMRRIMLDTSNMLFLGIPLDESAIVPKIQGYFDAWQALLLKPDIFFKFSWLHRKYEKSVKDLKDAMEILIEEKRHRI
STTEKLEDCMDFATELIFAERRGDLTKENVNQCILEMLIAAPDTMSVSVFFMLFLIAKHPQVEEAVMKEIQTVVGERDMRIDDMQKLKV
VENFIYESMRYQPVVDLVMRKALEDDVIDGYPVKKGTNIILNIGRMHRLEFFPKPNEFTLENFAKNVPYRYFQPFGFGPRACAGKYIAM
VMMKVTLVTLLKRFRVETLQGQCVEKIQKKNDLSLHPDETCKTRDLLEMIFIPRNSDKSLEH
>CYP19A2
MVLEMLNPMHYYNITSTVSGGVPVAAMAILLLTGFLLLVWNYKNTSSIPGPGYCLGIGPLISHCRFLWMGIGSASNYYNKMYGEFMRVW
ICGEETLIISKSSGVFHVMKHNHYVSRFGSKLGLQFIGMHEKGIIFNNNPVMWKAVRTYFMKALSGPGLVRMVTVCADSITKHLDRLEE
VRDEMGYVDVLTLMRRIMLDTSNMLFLGIPLDESAIVPKIQGYFDAWQALLLKPDIFFKFSWLHRKYEKSVKDLKDAMEILIEEKRHRI
STTEKLEDCMDFATELIFAERRGDLTKENVNQCILEMLIAAPDTMSVSVFFMLFLIAKHPQVEEAVMKEIQTVVGERDMRIDDMQKLKV
VENFIYESMRYQPVVDLVMRKALEDDVIDGYPVKKGTNIILNIGRMHRLEFFPKPNEFTLENFAKNVPYRYFQPFGFGPRACAGKYIAM
VMMKVTLVTLLKRFRVETLQGQCVEKIQKKNDLSLHPDETCKTRDLLEMIFIPRNSDKSLEH
Camelus dromedarius isolate Drom800 breed African chromosome 6, CamDro3
Exon 1 >NC_044516.1:15227834-15228019
Exon 2 >NC_044516.1:15232820-15232970
Exon 3 >NC_044516.1:15282899-15283053
Exon 4 >NC_044516.1:15279799-15279975
Exon 5 >NC_044516.1:15277261-15277375
Exon 6 >NC_044516.1:15275006-15275120
Exon 7 >NC_044516.1:15274406-15274568
Exon 8 >NC_044516.1:15272686-15272927
Exon 9 >NC_044516.1:15271237-15271506

Camelus ferus

>XP_032337094.1 aromatase [Camelus ferus]
MVLEMLNPIHYYNITSMVSEGVPVAAMAILLLTGFLLLVWNYEDTSSIPGPGYCLGIGPLISHCRFLWMGIGSASNYYNKMYGEFMRVW
ICGEETLIISKSSSMFHVMKHSHYISRFGSKLGLQFIGMHEKGIIFNNNPALWKAVRPFFTKALSGPGLVRMVTVCADSITKHLDRLEE
VRDEMGYVDVLTLMRRIMLDTSNMLFLGIPLDESAIVPKIQGYFDAWQALLLKPDIFFKFSWLHRKYEKSVKDLKDAMEILIEEKRHRI
STTEKLEDCMDFATELIFAERRGDLTKENVNQCILEMLIAAPDTMSVSVFFMLFLIAKHPQVEEAVMKEIQTVVGERDMRIDDMQKLKV
VENFIYESMRYQPVVDLVMRKALEDDVIDGYPVKKGTNIILNIGRMHRLEFFPKPNEFTLENFAKNVPYRYFQPFGFGPRACAGKYIAM
VMMKVTLVTLLKRFRVETLQGQCVEKIQKKNDLSLHPDETCKTRDLLEMIFIPRNSDKSLEH
>XP_006181378.2 LOW QUALITY PROTEIN: aromatase [Camelus ferus]
MVLEMLNPMHYYNITSTVSGGVPVAAMAILLLTGFLLLVWNYKNTSSIPGPGYCLGIGPLISHCRFLWMGIGSASNYYNKMYGEFMRVW
ICGEETLIISKSSGVFHVMKHNHYVSRFGSKLGLQFIGMHEKGIIFNNNPVMWKAVRTYFMKALSGPGLVRMVTVCADSITKHLDRLEE
VRDEMGYVDVLTLMRRIMLDTSNMLFLGIPLDESAIVPKIQGYFDAWQALLLKPDIFFKFSWLHRKYEKSVKDLKDAMEILIEEKRHRI
STTEKLEDCMDFATELIFAERRGDLTKENVNQCILEMLIAAPDTMSVSVFFMLFLIAKHPQVEEAVMKEIQTVVGERDMRIDDMQKLKV
VENFIYESMRYQPVVDLVMRKALEDDVIDGYPVKKGTNIILNIGRMHRLEFFPKPNEFTLENFAKNVPYRYFQPFGFGPRACAGKYIAM
VMMKVTLVTLLKRFRVETLQGQCVEKIQKNNDLSLHPDETCKTRDLLEMIFIPRNSDKSLEH

Vicugna vicugna

>CYP19A1
MVLEMLNPMHYYNITSMVSGGVPVAAMAILLLTAFLLLVWNYEDTSSIPGPGYCLGIGPLISHCRFLWMGIGSASNYYNKMYGEFMRVW
ICGEETLIISKSSSMFHVMKHSHYISRFGSKLGLQFIGMHEKGIIFNNNPALWKAVRPFFTKALSGPGLVRMVTVCADSITKHLDRLEE
VRDEMGYVDVLTLMRRIMLDTSNMLFLGIPLDESAIVAKIQGYFDAWQALLLKPDIFFKFSWLHRKYEKSVKDLKDAMEILIEEKRHRI
STAEKLEDCMDFATELIFAERRGDLTKENVNQCILEMLIAAPDTMSVSVFFMLFLIAKHPQVEEAVMKEIQTVVGERDMRIDDMQKLKV
VENFIYESMRYQPVVDLVMRKALEDDVIDGYPVKKGTNIILNIGRMHRLEFFPKPNEFTLENFAKNVPYRYFQPFGFGPRACAGKYIAM
VMMKVTLVTLLKRFRVETLQGHCVEKIQKKNDLSLHPDETCKTRDLLEMIFIPRNSDKSLEH
Vicugna vicugna mensalis isolate CD3-2017 contig3610, whole genome shotgun sequence
Exon 1 >PNXW01003610.1:279-465
Exon 2 >PNXW01012561.1:529-679
Exon 3 >PNXW01007519.1:103-257

Exon 4 >PNXW01007519.1:3532-3708
Exon 5 >PNXW01007519.1:5595-5709
Exon 6 >PNXW01007519.1:7852-7966
Exon 7 >PNXW01007519.1:8402-8564
Exon 8 >PNXW01007519.1:10128-10369
Exon 9 >PNXW01007519.1:11552-12868
>CYP19A2 (incomplete)
MVLEMLNPMHYYNITSMVSGGVPVAAMAILLLTAFLLLVWNYKNKSSIP…exon2_missing…SSAVFHVMKHNHYVSRFGSKLGLQF
IGMHDKGIIFNNNPVMWKAVRTYFMK…exon4,5,6,7,8,9_missing
Vicugna vicugna mensalis isolate CD3-2017 contig12560,whole genome shotgun sequence
Exon 1 >PNXW01012560.1:19919-20104
Exon 2 missing
Exon 3 >PNXW01023566.1:2053-2207
Exon 4, 5, 6, 7, 8, 9 missing

Hippopotamus amphibius

>XP_057577862.1 aromatase [Hippopotamus amphibius kiboko]
MVLEVLNPMHYNITSTVSEGVPVASIAILLLTGFLLFVWNYEDTSSIPGPGYFLGIGPLISHCRFLWMGIGSACNYYNKMYGEFMRVWI
CGEETLIISKSSSMFHVMKHSHYTSRFGSKLGLQCIGMHEKGIIFNNNPALWKAVRPFFTKALSGPGLVRMVTICADSITKHLDRLEEV
RNELGYVDVLTLMRRIMLDTSNILFLGIPLDESAIVVKIQGYFDAWQALLLKPDIFFKISWLYRKYEKSVKDLKDAMEILIEEKRHRIS
TAEKLEDCMDFATELIFAEKRGDLTRENVNQCILEMLIAAPDTMSVSVFFMLFLIAEHPHVEEAIVKEIQTVVGERDIRIDDMQKLKVV
ENFIYESMRYQPVVDLVMRKALEDDVIDGYPVKKGTNIILNIGRMHRLEFFPKPNEFTLDNFAKNVPYRYFQPFGFGPRACAGKYIAMV
MMKVILVTLLRRFRVQTLQGRCVETMQKKNDLSLHPDETGDLLEMIFIPRNSDKCLER
>XP_057578169.1 aromatase-like [Hippopotamus amphibius kiboko]
MVLEVLNPMHYNITSTVSEGAPVASIAILLLTGFLLFVWTYKNTSSIPGPGYFLGIGPLISHCRFLWMGIGSACNYYNKMYGEFMRVWI
CGEETLIISKSSGVFHVMKHSHYTSRFGSKLGLQCIGMHEKGIIFNNNPELWKTARTYFMKALSGPGVVRMVTICADSITKHLDRLEEV
RNELGYVDVLTLMRRIMLDTSNMLFLGIPLDESAIVVKIQGYFDAWQALLLKPDIFFKISWLYRKYEKSVKELKDEMEILTEKKRRKIF
TAEKLEDSMDFATELILAEKRGDLTRENVNQCILEMLIAAPDTMSISVFFMLFLIAKHPQVEEAIMKEIQTVVGERDIKNDDIQKLKVV
ENFIYESMRYQPVVDLVMRKALEDDVIDGYPVKKGTNIILNIGRMHRLEFFPKPNEFTLDNFAKNVPYRYFQPFGFGPRACAGKYIAMV
MMKVILVTLLRRFRVQTLQGRCVETMQKKNDLSLHPDETGDLLEMIFIPRNSDKCLER

Physeter catodon

>XP_023972459.1 aromatase [Physeter catodon]
MVLEVLNSVHYNVTSMVTEVAPVASIAILLFTGFLLLVWNYEDTSSIPGPGYFLGIGPLISHCRFLWMGIGSACNYYNKMYGEFMRVWI
CGEETLIISKSSSMFHIMKHSHYTSRFGSKLGLQCIGMHEKGIIFNNNPALWKAVRPFFTKALSGPGLVRMVTVCADSITKHLDRLEEV
CNELGYVDVLTLMRRIMLDTSNKLFLGIPLDERAIVVKIQGYFDAWQALLLKPDIFFKISWLCRKYEKSVKDLKDAMEILIEEKRQRIS
TAEKLEDCMDFATELIFAEKRGDLTRENVNQCILEMLIAAPDTMSVSVFFMLFLIAKHPQVEEAIMKEIQTVVGERDIRIDDMQKLKVV
ENFIYESMRYQPVVDLVMRKALEDDVIDGYPVKKGTNIILNIGRMHRLEFFPKPNEFTLENFARNVPYRYFQPFGFGPRACAGKYIAMV
MMKVTLVTLLRRFHVQTLQGRCVEKMQKKHDLSLHPDETSDLLGMIFIPRNSDNCLDH
>XP_023972897.1 aromatase-like [Physeter catodon]
MVLEVLNSVHYNVTSMVSEVAPVASIAIMLLTGFLLLVWNYKNISSISGPGYFLGIGPLISYCRFLWMGNGSACNYYNKTYGEFVRVWI
YGEETLIISKSSSVFHVMKHNHYTSRFGSKLGLQCIGMHEKGIIFNNNPVLWKAVRTYFMKALSGPSLVRMVTVCADSITKHLDRLEEV
RNELGYVDVLTLMRCIMLDTSNKLFLGIPLDERAIVVKIQGYFDAWQALLLKPDIFFKISWLCRKYEKSVKELKDEMEILVEKKRRRIF
IAEKLEDSMDFATELILAEKRGDLTRENVNQCVLEMLIAAPDTMSVSVFFMLLLIAKHPQVEEAIMKEIQTVVGERDIRIDDIQKLKVV
ENFIYESMRYQPVVDLVMRKALEDDVIDGYPVKKGTNIILNIGRMHRLEFFPKPNEFTLENFAKNVPYRYFQPFGFGPRACAGKYIAMV
MMKVTLVTLLRRFHVQTLQGQWVENMQKKNDLSLHPDETSDLLGMIFIPRNSDKCLDH

Tursiops truncatus

>XP_033708501.1 aromatase [Tursiops truncatus]
MVLEVLNPRHYNITSMVTEVAPVASIAILLLTGFLLLVWNYEDTSSIPGPGYFLGIGPLISHCRFLWMGIGSTCNYYNKTYGEFVRVWI
CGEETLIISKSSSMFHIMKHSHYTSRFGSKLGLQCIGMHEKGIIFNNNPALWKAVRPFFTKALSGPGLVRMVTVCADSITKHLDRLEEV
RNELGYVDVLTLMRRIMLDTSNKLFLGIPLDERAIVVKIQGYFDAWQALLLKPDIFFKISWLRRKYEKSVKDLKDAMEILIEEKRQRIS
TAEKLEDCMDFATELIFAEKRGDLTKENVDQCILEMLIAAPDTMSVSVFFMLFLIAKHPQVEEAIMKEIQTVVGERDIRIDDMQKLKVV
ENFIYESMRYQPVVDLVMRKALEDDVIDGYPVKKGTNIILNIGRMHRLEFFPKPNEFTLENFARNVPYRYFQPFGFGPRACAGKYIAMV
MMKVTLVTLLRSFHVQTLQGRCIEKMQKKNDLSLHPDETSDLLGMIFIPRNSDKCLDH

>XP_033708499.1 aromatase-like [Tursiops truncatus]
MVLEVLNSVHYNIISMVTEVVPVASIAILLLTGFLLLVWNYKNTSSIPGPGYFLGIGPLISYCRFLWMGNGSACNYYNKTYGEFVRVWI
YGEETLIISKSSSVFHVMKHSHYTSRFGSKLGLQCIGMHEKGVIFNNNPVLWKAVRTYFMKALSGSGLVRTVTVCADSITKHLDRLEEV
RNESGYVDVLTLVRCIMLDTSNKLFLGIPLNERAIVVKIQGYFDAWQALLLKPDIFFKISWLRRKYEKSVEELKEEMEILVEKKRRRIF
IAEKLEDSMDFTTELILAEKRGDLTRENVNQCVLEMLIAAPDTMSVSVFFMLFLIAKHPQVEEAIMKEIQTVVAEREIRIDDMQKLKVV
ENFIYESMRYQPVVDLVMRKALEDDVIDGYPVKKGTNIILNIGRMHRLEFFPKPNEFTLENFAKNVPYRYFQPFGFGPRACAGKYIAMV
MMKVILVTLLRRFHMQTLHGQCVEKMQKKNDLSLQPDETSDLLGMIFIPRNSDKCLDH

Balaenoptera musculus

>XP_036699724.1 aromatase [Balaenoptera musculus]
MVLEVLNPRHYNITSMVSEVMPIASIAILLLTGFFLLVWNYEDTSSIPGPGYFLGIGPLISHCRFLWMGIGSACNYYNKTYGEFMRVWI
CGEETLIISKSSSMFHIMKHSHYTSRFGSKLGLQCIGMHEKGIIFNNNPALWKAVRPFFTKALSGPGLVRMVTVCADSITKHLDRLEEV
RNELGYVDVLTLMRRIMLDTSNKLFLGIPLDERAIVVKIQGYFDAWQALLLKPDIFFKISWLCRKYEKSVKDLKDAMEILIEEKRQRIS
TAEKLEDCMDFATELIFAEKRGDLTRENVNQCILEMLIAAPDTMSVSVFFMLFLIAKHPQVEEAIMKEIQTVVGERDIRIDDMQKLKVV
ENFIYESMRYQPVVDLVMRKALEDDVIDGYPVKRGTNIILNIGRMHRLEFFPKPNEFTLENFARNVPYRYFQPFGFGPRACAGKYIAMV
MMKVTLVTLLRRFHVQTLQGRCVEKMQKKNDLSLHPDETSDLLGMIFIPRNSDKCLGH
>XP_036699727.1 aromatase-like [Balaenoptera musculus]
MVLEVLNSVHYNVTSMVSEVAPVASIAILLLTGFLLLVWNYKNTSSIPGPGYFLGIGPLISYCRFLWMGIGSACNYYNKTYGEFVRVWI
YGEETLIISKSSSVFHVMKHNHYTSRFGSKLGLQCIGMHEKGIIFNNNPVLWKAARTYFMKALSGPGLVRMVTVCADSITKHLDGLEEV
RNELGYVDVLTLMRRIMLDTSNKLFLGIPLDERAIVVKIQGYFDAWQALLLKPDIFFKISWLCRKYEKSVKELKDEMEILVEKKRRRIF
IAEKLEDSMDFATELILAEKRGDLTRENVNQCVLEMLIAAPDTMSVSVFFMLLLIAKHPQVEEAIMKEIQTVVGERDIRIDDIQTLKVV
ENFIYESMRYQPVVDLVMRKALEDDVIDGYPVKRGTNIILNIGRMHRLEFFPKPNEFTLENFAKNVPYRYFQPFGFGPRACAGKYIAMV
MMKVTLVTLLRRFHVQTLQGQCVEKMQKKNDLSLHPDETSDLLGMIFIPRNSDKCLDH
>XP_036699726.1 aromatase-like [Balaenoptera musculus]
MVLEVLNPRHYNVTSMVTEVAPVASIAILLLTGFLLLVWNYKNTSSIPGPGYFLGIGPLISYCRFLWMGIGSACNYYNKTYGEFMRAWI
YGEETVIISKSSSVFHVMKYNHYTSRFGSKLGLQCIGMHEKGIIFNNNPVLWKAARTYFMKALSGPGLVRMVTVCADSITKHLDRLEEV
RNELGYVDVLTLMRRIMLDTSNKLFLGIPLDERAIVVKIQGYFDAWQALLLKPDIFFKISWLCRKYEKSVKELKDEMEILVEKKRRRIF
IAEKLEDSMDFATELILAEKRGDLTRENVNQCVLEMLIAAPDTMSVSVFFMLLLIAKHPQVEEAIMKEIQTVVGERDIRNDDIQKLKVM
ENFIYESMRYQPVLDLVMRKALEDDVIDGYPVKKGTNIILNIGRMHRLEFFPKPNEFTLENFAKNVPYRYFQPFGFGPRACVGKYIAMV
MMKVTLVTLLRRFHVQTLQGQCVEKMQKKHDLSLQPDETSDLLGMIFIPRNSDKCFDQ

Tragulus kanchil

>CYP19A1
MVLEVLSPRHYNITSLVSEAVPAASTAILLLAGVLLFVWNYKDTSSIPGPSYFLGIGPLISHCRFLWMGIGSACNYYNKMYGEFTRVWI
CGEETLIISRSSSMFHVMKHSHYISRFGSKLGLQFIGMHEKGIIFNNNPALWKAVRPFFTKALSGPGLVRMVTICADSITKHLDRLEEV
RNDSGYVDVLTLMRRIMLDTSNTVFLGIPLDESAIVVKIQGYFDAWQALLLKPDIFFKISWLCRKYEKSVKDLKDAMEILIEEKRHRIS
TAEKLEDCMDFATELIFAEKRGELTRENVNQCILEMLIAAPDTMSVSVFFMLFLIAKHPQVEEAIMKEIQTVVGERDIRIDDMQKLKVV
ESFIYESMRYQPVVDLVMRKALEDDVIDGYPVKKGTNIILNIGRMHRLEFFPKPNEFTLENFAKNVPYRYFQPFGFGPRACAGKYIAMV
MMKVILVTLLKRFHVQTLQGRCVEKMQKKNDLSLHPDETSDLLEMIFTPRNSDKCIAY

Tragulus kanchil isolate TJ002 scaffold13, whole genome shotgun sequence
Exon 1 >JAIOJX010000024.1:167960361-167960538
Exon 2 >JAIOJX010000024.1:167950789-167950939
Exon 3 >JAIOJX010000024.1:167932901-167933055
Exon 4 >JAIOJX010000024.1:167927746-167927922
Exon 5 >JAIOJX010000024.1:167924137-167924251
Exon 6 >JAIOJX010000024.1:167919253-167919367
Exon 7 >JAIOJX010000024.1:167858639-167858801
Exon 8 >JAIOJX010000024.1:167913626-167913867
Exon 9 >JAIOJX010000024.1:167911276-167912432
>CYP19A2
MALEVLSLLHSGVTSLVAKAVPVASTAILLLAGVLLLTWNYKNTASIPGPSYFLGIGPLISHCRFLWMGIGSACNYYNKMYGEFTRVWI
CGEETIITSRSSSLFHVMKHNHYISRFGSKLGLQIIGMHENGIIYNNNPVLWKAVRTYFMKALSGPGLVRMVTICADSITKHLDRLEEV
RNDSGYVDVLTLMRRIMLDTSNTVFLGIPLDESAIVVKIQGYFDAWQALLLKPDIFFKIPWLCRKYEKSVKDLKAELEILIEKKRHRIS
IAEKLEDSMDFATELILAEKRGELTRENVNQCILEMLIAAPDTMSVSVFFMLFLIAKHPQVEEAIMKEIQTVVGERKIKNEDIQKLNVL

ENFIYESMRYQPVVDLVMRKALEDDVIDGYPVKKGTNIILNIGRMHRLEFFPKPNEFTLENFAKNVPYRYFQPFGFGPRACAGKYIAMV
MMKVVLATLLRRFHVQTLRGHCVEKMQKNNDLSLHPDETTDLLEMIFTPRNSDKCIEC
Tragulus kanchil isolate TJ002 scaffold13, whole genome shotgun sequence
Exon 1 >JAIOJX010000024.1:167877983-167878165
Exon 2 >JAIOJX010000024.1:167875848-167875998
Exon 3 >JAIOJX010000024.1:167869730-167869884
Exon 4 >JAIOJX010000024.1:167865527-167865703
Exon 5 >JAIOJX010000024.1:167861856-167861970
Exon 6 >JAIOJX010000024.1:167861139-167861253
Exon 7 >JAIOJX010000024.1:167858639-167858801
Exon 8 >JAIOJX010000024.1:167854836-167855077
Exon 9 >JAIOJX010000024.1:167847902-167851326

Tragulus javanicus

>CYP19A1
MVLEVLSPRHYNITSLVSEAVPAASTAILLLAGVLLFVWNYKDTSSIPGPSYFLGIGPLISHCRFLWMGIGSACNYYNKMYGEFTRVWI
CGEETLIISRSSSMFHVMKHSHYISRFGSKLGLQFIGMHEKGIIFNNNPALWKAVRPFFTKALSGPGLVRMVTICADSITKHLDRLEEV
RNDSGYVDVLTLMRRIMLDTSNTVFLGIPLDESAIVVKIQGYFDAWQALLLKPDIFFKISWLCRKYEKSVKDLKDAMEILIEEKRHRIS
TAEKLEDCMDFATELIFAEKRGELTRENVNQCILEMLIAAPDTMSVSVFFMLFLIAKHPQVEEAIMKEIQTVVGERDIRIDDMQKLKVV
ESFIYESMRYQPVVDLVMRKALEDDVIDGYPVKKGTNIILNIGRMHRLEFFPKPNEFTLENFAKNVPYRYFQPFGFGPRACAGKYIAMV
MMKVILVTLLKRFHVQTLQGRCVEKMQKKNDLSLHPDETSDLLEMIFTPRNSDKCIAY
Tragulus javanicus isolate US108 ScTR3Pl_527;HRSCAF=35978_0, whole genome shotgun sequence
Exon 1 >PVHZ020000536.1:1033378-1033555
Exon 2 >PVHZ020000536.1:1039206-1039355
Exon 3 >PVHZ020000536.1:1054814-1054969
Exon 4 >PVHZ020000536.1:1059787-1059962
Exon 5 >PVHZ020000536.1:1062747-1062862
Exon 6 >PVHZ020000536.1:1065477-1065591
Exon 7 >PVHZ020000536.1:1066248-1066410
Exon 8 >PVHZ020000536.1:1068184-1068425
Exon 9 >PVHZ020000536.1:1069617-1070955

Bos taurus

>NP_776730.1 aromatase [Bos taurus]
MLLEVLNPRHYNVTSMVSEVVPIASIAILLLTGFLLLVWNYEDTSSIPGPSYFLGIGPLISHCRFLWMGIGSACNYYNKMYGEFMRVWV
CGEETLIISKSSSMFHVMKHSHYISRFGSKLGLQFIGMHEKGIIFNNNPALWKAVRPFFTKALSGPGLVRMVTICADSITKHLDRLEEV
CNDLGYVDVLTLMRRIMLDTSNMLFLGIPLDESAIVVKIQGYFDAWQALLLKPDIFFKISWLCRKYEKSVKDLKDAMEILIEEKRHRIS
TAEKLEDSIDFATELIFAEKRGELTRENVNQCILEMLIAAPDTMSVSVFFMLFLIAKHPQVEEAIIREIQTVVGERDIRIDDMQKLKVV
ENFINESMRYQPVVDLVMRKALEDDVIDGYPVKKGTNIILNLGRMHRLEFFPKPNEFTLENFAKNVPYRYFQPFGFGPRACAGKYITMV
MMKVVLVTLLRRFHVQTLQGRCVEKMQKKNDLSLHPDETRDRLEMIFTPRNSDKCLER

Ovis aries

>NP_001116472.1 aromatase [Ovis aries]
MLLEVLNPRHYNVTSMVSEVVPIASIAILLLTGFLLLVWNYEDTSSIPGPSYFLGIGPLISHCRFLWMGIGSACNYYNKMYGEFMRVWV
CGEETLIISKSSSMFHVMKHSHYISRFGSKLGLQFIGMHEKGIIFNNNPALWKAVRPFFTKALSGPGLVRMVTICADSITKHLDRLEEV
CNDLGYVDVLTLMRRIMLDTSNILFLGIPLDESAIVVKIQGYFDAWQALLLKPDIFFKISWLCRKYEKSVKDLKDAMEILIEEKRHRIS
TAEKLEDCIDFATELIFAEKRGELTKENVNQCILEMLIAAPDTMSVSVFFMLFLIAKHPQVEEAMMREIQTVVGERDIRIDDMQKLKVV
ENFINESMRYQPVVDLVMRKALEDDVIDGYPVKKGTNIILNLGRMHRLEFFPKPNEFTLENFAKNVPYRYFQPFGFGPRACAGKYIAMV
MMKVILVTLLRRFHVQTLQGRCVEKMQKKNDLSLHPDETSDRLEMIFIPRNSDKCLEC

Capra hircus

>NP_001272676.1 aromatase [Capra hircus]

MLLEVLNPRHYNVTSMVSEVVPIASIAILLLTGFLLLVWNYEDTSSIPGPSYFLGIGPLISHCRFLWMGIGSACNYYNKMYGEFMRVWV
CGEETLIISKSSSMFHVMKHSHYISRFGSKLGLQFIGMHEKGIIFNNNPALWKAVRPFFTKALSGPGLVRMVTICADSITKHLDRLEEV
CNDLGYVDVLTLMRRIMLDTSNILFLGIPLDESAIVVKIQGYFDAWQALLLKPDIFFKISWLCRKYEKSVKDLKDAMEILIEEKRHRIS
TAEKLEDCIDFATELIFAEKRGELTKENVNQCILEMLIAAPDTMSVSVFFMLFLIAKHPQVEEAMMREIQTVVGERDIRIDDMQKLKVV
ENFINESMRYQPVVDLVMRKALEDDVIDGYPVKKGTNIILNLGRMHRLEFFPKPNEFTLENFAKNVPYRYFQPFGFGPRACAGKYIAMV
MMKVILVTLLRRFHVQTLQGRCVEKMQKKNDLSLHPDETSDRLEMIFIPRNSDKCLEC

Oryx dammah

>XP_040082447.1 aromatase [Oryx dammah]
MLLEVLNPRHYNVTSMVSEVVPIASIAILLLTGFLLLVWNYEDTSSIPGPSYFLGIGPLISHCRFLWMGIGSACNYYNKMYGEFMRVWV
CGEETLIISKSSSMFHVMKHSHYISRFGSKLGLQFIGMHEKGIIFNNNPALWKAVRPFFTKALSGPGLVRMVTICADSIIKHLDRLEEV
RNDLGYVDVLTLMRRIMLDTSNMLFLGIPLDESAIVVKIQGYFDAWQALLLKPDIFFKISWLCRKYEKSVKDLKDAMEILIEEKRHRIS
TAEKLEDCIDFATELIFAEKRGELTRENVNQCILEMLIAAPDTMSVSVFFMLFLIAKHPQVEEAIMREIQTVVGERDIRIDDMQKLKVV
ENFINESMRYQPVVDLVMRKALEDDVIDGYLVKKGTNIILNLGRMHRLEFFPKPNEFTLENFAKNVPYRYFQPFGFGPRACAGKYIAMV
MMKVILVTLLRRFHVQTLQGRCVEKMQKKNDLSLHPDETSDRLEMIFIPRNSDKCLER
