# Supplementary figures and images for: Charged Amino Acids in the Transmembrane Helix Strongly Affect the Enzyme Activity of Aromatase
Source: Int J Mol Sci. 2024 Jan 24;25(3):1440. doi: 10.3390/ijms25031440 (PMC10855386; doi:10.3390/ijms25031440)

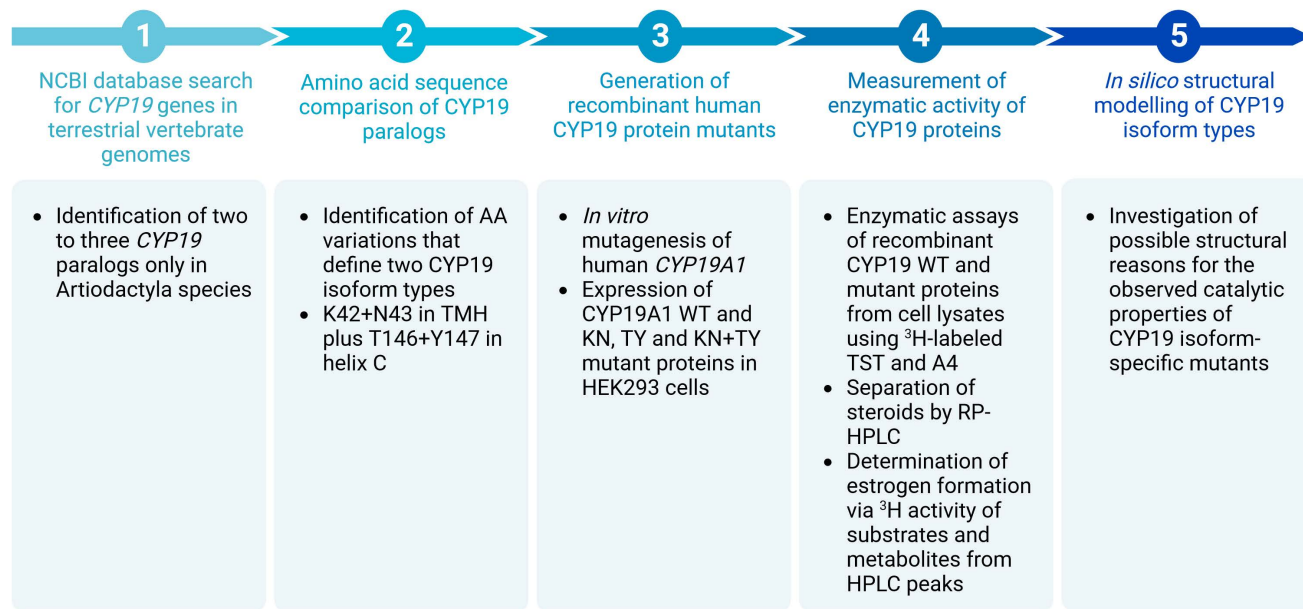

**Figure S3.** Flow chart illustrating methodology and study design. Created with BioRender.com

Supplement: Supplementary file 1 [file ijms-25-01440-s001.zip › Figure S3_new.pdf]
